# Violence against Women and Gastroschisis: A Case-Control Study

**DOI:** 10.3390/ijerph10105178

**Published:** 2013-10-17

**Authors:** Juan Antonio Ortega-García, Offie P. Soldin, Miguel Felipe Sánchez-Sauco, Alicia Cánovas-Conesa, Virtudes Gomaríz-Peñalver, Diana Carolina Jaimes-Vega, Joseph E. Perales, Alberto Cárceles-Alvarez, Maria Teresa Martínez-Ros, Daniel Ruiz

**Affiliations:** 1 Paediatric Environmental Health Speciality Unit, Department of Paediatrics, Hospital Clinic University Virgen of Arrixaca, Murcia, 30120, Spain; E-Mails: miguel@pehsu.org (M.F.S.-S.); alicia@pehsu.org (A.C.-C.); virtudes@pehsu.org (V.G.-P.); diana@pehsu.org (D.C.J.-V.); josephed@hotmail.com (J.E.P.); alberto@pehsu.org (A.C.-A.); druiz7012@gmail.com (D.R.); 2 Georgetown University Medical Center, Washington, DC 20007, USA; E-Mail: os35@georgetown.edu; 3 Direction of Healthcare System, Murcia Health Service, Regional Ministry of Health, Murcia, 30008, Spain; E-Mail: mariat.martinez4@carm.es

**Keywords:** gastroschisis, risk factors, domestic violence, health promotion, case-control study

## Abstract

*Background*: Gastroschisis, a birth defect characterized by herniated fetal abdominal wall, occurs more commonly in infants born to teenage and young mothers. Ischemia of the vascular vitelline vessels is the likely mechanism of pathogenesis. Given that chronic stress and violence against women are risk factors for cardiovascular disease we explored whether these may represent risk factors for gastroschisis, when they occur during pregnancy. A case-control study was conducted, with 15 incident cases of children born with gastroschisis in the Region of Murcia, Spain, from December 2007 to June 2013. Forty concurrent controls were recruited at gestation weeks 20–24 or *post-partum*. All mothers of cases and controls completed a comprehensive, in-person, ‘green sheet’ questionnaire on environmental exposures. *Results*: Mothers of children with gastroschisis were younger, smoked more cigarettes per week relative to controls, were exposed to higher amounts of illegal drugs, and suffered from domestic violence more frequently than the controls. Multivariable logistic regression analysis highlights periconceptional ‘gender-related violence’ (OR: 16.6, 95% CI 2.7 to 101.7) and younger maternal age (OR 1.1, 95% CI 1.0–1.3). *Conclusions*: Violence against pregnant women is associated with birth defects, and should be studied in more depth as a cause-effect teratogenic. Psychosocial risk factors, including gender-based violence, are important for insuring the health and safety of the pregnant mother and the fetus.

## 1. Introduction

Gastroschisis is the most common of the abdominal wall closure defects. The incidence of this pathology has been increasing in recent years, and has reached the incidence of 4–5 cases per 10,000 for all live births [1,2]. The primary risk factor is young maternal age, with the majority of babies born with gastroschisis born to young mothers who are in their teens or early twenties in their first pregnancy. The exact cause for the development of these defects is still unknown; however, it has been speculated that possible etiologic factors include genetic factors, lifestyle and habits of young women, early exposure to legal and illegal drugs, toxic chemicals and adverse nutritional status during the pregnancy [1,2,3]. Gastroschisis remains an epidemiologic and pathogenetic dilemma, with the thought that genetics do not play a significant role in its etiology. Nevertheless, the genetic susceptibility should be further investigated because it may have a greater role in the etiology of gastroschisis than currently appreciated [4]. Table 1 shows the known environmental risk factors of gastroschisis [1,3,5,6,7,8,9,10,11,12,13,14,15,16,17,18,19,20,21,22]. One proposed mechanism of pathogenesis of this malformation seems to be related to the intrauterine interruption of the omphalomesenteric vessel flow and the secondary ischemic injury in the first weeks of pregnancy [23,24].

**Table 1:** Environmental Risk factors studied for Gastroschisis.

Risk factors	Description	OR (95% CI) **
Consistent evidence		
Early maternal age	14–19 years 12–15 years	7.2 (4.4–11.2) [5,6,7,8,9] 4.2 (2.5–7.0) [10]
Limited evidence		
Early paternal age	20–24 years per ten years younger	1.5 (1.1–1.9) [11] 1.6 (1.0–2.4) [12]
Race [12]	Hispanics > white Non-hispanics > black	
Low incomes Level of education	<10,000$ No relation 5.7	4.5 (1.4–14.4) [7]
Drugs: Aspirin/silicates	Metaanalysis	2.4 (1.4–3.9) [14]
Nasal descongestant	Phenilpropanoamine Pseudoephedrine	10.0 (1.2–85.6) [15] 2.1 (0.8–5.5) [15]
Legal and illegal drugs		
Alcohol	Maternal intake during 1^st^ trimester Any excessive alcohol intake 6 or more drinks per week 5 or more drinks at a time	2.4 (1.4–3.7) [7] 2.3 (1.1–4.9) [7] 2.9 (1.1–7.4) [5] 3.2 (1.5–6.7) [5]
Smoking during pregnancy	Numerous published researches with significant relation	Rango 1.2–2.1 [16]
Illegal Drugs	Any drugs Cocaine, amphetamines or ecstasy	2.2 (1.2–4.3) [17] 3.3 (1.0–10.5) [17]
Nutritional Factors		
BMI *	BMI < 18.1 kg/m^2^ BMI > 28.3 kg/m^2^ BMI < 18.5 kg/m^2^ Overweigth 25–30 kg/m^2^ Obese ≥ 30 kg/m^2^ IMC ≤ 30 kg/m^2^ BMI < 18.5 kg/m^2^	3.2 (1.4–7.4) [18] 0.2 (0.04–0.8) [18] 2.0 (1.1–3.7) [17] 0.3 (0.2–0.7) [17] 0.3 (0.1–0.8) [17] 0.2 (0.1–0.3) [19] 0.9 (0.6–1.2) [19]
Specific nutrients	Low carotene Low sugar High nitrosamines Monounsaturated fatty acids (oleic acid, g) Vegetable intake (rations/week)	4.3 (1.9–9.8) [20] 3.3 (1.4–7.6) [20] 2.6 (1.3–5.4) [20] 0.7 ( 0.6–0.9) [21] 0.7 (0.4–1.0) [21]
Ionising radiation	During the first trimester of pregnancy	2.5 (1.2–5.5) [15]
Solvents (occupations and hobbies)	Solvents (aromatic hydrocarbons and aliphatics)	6.3 (2.2–18.3) [15]
Short cohabitation Change of paternity	Short cohabitation (<1 year) Multiparous with short cohabitation that had changed partner	2.4 (1.5–3.7) [9] 8.7 (2.1–21.1) [9]
Genitourinary infections	Self-reported urinary tract infection plus sexually transmitted infection	4.0 (1.4–11.6) [22]
Factors without significant relation		
Coffee [5]	No significant correlation has been found to oral contraceptives neither antihistamines	
Other drugs [7]		
Upper respiratory infections, fever and allergies [5,6]		

* BMI = body mass index, ** CI = confidence intervals; Sources for all environmental risk factors listed in Table 1 are in references [1,3,5,6,7,8,9,10,11,12,13,14,15,16,17,18,19,20,21,22].

Pregnancy is a unique condition in a couple’s life, with significant changes in the physiology of the mother and many added demands. At that time, environmental factors (physical, chemical, biological, and psychosocial) play a role in both intrauterine development and fetal circulation. Thus, malnutrition, chronic infections, exposure to legal and illegal drugs, exposure to heavy metals, maternal mental status and her exposure to stressful environments with violence or lack of affection are all known risk factors that hamper optimal fetal development [25]. A father who is fulfilled in his affective needs and has an empathic attitude is able to provide assurance and support to both the mother and newborn. Recent research considers early paternal age, changes in paternity, and a short cohabitation duration of the couple as additional risk factors for gastroschisis [9,26]. Teenage pregnancy tends to trigger inequalities in gender relations and increases the vulnerability of women, could often result in various forms of social subordination. In 1993, The General Assembly of the United Nations defined violence against women as any act of gender-based violence that results, or may result in physical, sexual or psychological harm or suffering to women, including threats of such acts, coercion or arbitrary deprivation of liberty, regardless of whether this occurs in public or private life [27]. The most common type of violence against women worldwide is physical, emotional, or sexual abuse of women by their male partners or former intimate partners [28]. Suffering violence in a relationship has serious short and long term consequences for women’s health [28,29]. Adopting a gender-specific perspective when approaching the etiology of gastroschisis is sensible because stress and gender-related violence are known cardiovascular risk factors [30]. The aim of this study is to analyze the risk factors for gastroschisis, given special attention to psychosocial factors related to periconceptional gender violence.

## 2. Materials and Methods

A population-based case-control study was conducted in the Region of Murcia from December 2007 to June 2013. The Region of Murcia is located in south-eastern Mediterranean Spain with 14,500 newborns born in 2012. Cases of gastroschisis included all of the incidental fetuses, stillbirths, or newborns diagnosed with gastroschisis during pregnancy or the neonatal period during the time frame of this study in the Region of Murcia. The University Hospital Virgen of Arrixaca registration has centralized healthcare in regional reference units of Pediatric Surgery, Fetal Medicine, and Environmental Health, and The University Hospital Virgen of Arrixaca registers 100% of diagnosed patients and has full access to their medical histories. All suspected cases of prenatal diagnosis were confirmed postnatally. Fifteen incident cases were diagnosed; twelve of them during pregnancy while three were in the neonatal period.

Forty healthy controls, without malformations, were recruited concurrently at the time when the cases were recruited matched by reference health area and the time of diagnosis (pregnancy or postpartum) of the corresponding case. Those individuals recruited during 20–24 weeks of pregnancy, the fetal outcome was verified upon delivery. In the controls, the newborns or fetuses born with malformations were excluded from the study. 

The fathers and mothers were referred to the Environmental Pediatric Consultation, where the study was explained to them and they were invited to participate. Informed consent was obtained from both parents for all cases and controls. In addition, parents of mothers who were younger than sixteen were also asked to complete the informed consent form. The study was approved by the institutional review board of the University Hospital Virgen of Arrixaca. 

We have defined the gender violence variable based on the following criteria: Traumatic separation of the couple at the first trimester of pregnancy, and/or partner imprisoned with a history of abuse, and/or physical abuse (objectified or complaint), and/or maternal anxiety attack related to abuse by their male partner (tested with an accompanying case report). Other psychosocial variables included the search getting for pregnancy, cohabitation time before the last menstrual period, leaving home at first trimester of pregnancy, and the use of any contraceptive. All data collection interviews were conducted in person, with both parents present, and also with each parent individually, in order to correctly collect the related gender violence variables [31]. These factors and other environmental data and health-related information were collected using the parents’ environmental reproductive health questionnaire. This document contains concise and simple questions that aim to detect the absence or presence of evidence-based risk factors associated with the disease [1,21,32,33,34]. The interviews were conducted by the trained staff of our environmental health and risk team. They knew who they were interviewing cases or controls. However, we think, that the strength of our study is that, unlike most studies, our patients’ information was collected through personal interviews conducted by the specially trained personnel. Therefore, the possibilities for error, recall bias, or missing information were minimized and comparable in the two groups. The general variables of the questionnaire for gastroschisis are listed in Table 2. Other variables considered in the study were: parental age, exposure to legal and illegal drugs, parental education, income level, weight gain and body mass index in early pregnancy. The medical consultation lasts approximately 15–25 min. 

**Table 2:** Items in the ‘green sheet’ questionnaire for gastroschisis cases.

Genealogical factors:	Gender Race/ethnicity Family History Genealogical tree
Reproductive medical History	Gynecological History Hormonal Therapy
Enviromental Factors	Socioeconomic level Home exposure The community Medical History of Ionizing Radiation exposure Illegal and legal drugs use Home remedies/supplements and herbals Medications Exposure due to occupation Hobbies

Univariate analysis was performed (Chi-squared y *t*-test). A model was constructed with significant variables of the univariate analysis by logistic multivariate regression with introduction, forward and backward and inclusion of significant variables 2 × 2, 3 × 3 and 4 × 4 in the univariate analysis to control the potential confounder factors. Effects were considered statistically significant at a *P* value < 0.05 and Odds Ratio (OR), in which confidence interval (CI) was 95%, does not include one.

## 3. Results

The case-controls participation rate was 100%. Mothers and fathers of children with gastroschisis were young and were more likely to be exposed to tobacco and illegal drugs than controls. Mothers of children with gastroschisis were not seeking to get pregnant, and demonstrate living in a traumatic stress environment with signs of gender violence. Table 3 demonstrates the differences in the variables studied between cases and controls. Univariate analysis revealed the significant variables, namely, gender violence and maternal age, in close association with gastroschisis. 

**Table 3:** Sociodemographics and confounding variables in cases and controls.

	Cases		Controls					
	N (%)	Average (IC95%)	N (%)	Average (IC95%)	χ²	*t* -test	Univariate OR (IC95%)
Sociodemographics							
Age							
Mother *		21.6 (18.8–24.3)		29.2 (27.1–31.2)		<0.01	1.2 (1.1–1.4)
Father *		26.2 (23.0–29.4)		32.0 (30.0–34.0)		<0.01	1.1 (1.0–1.3)
Level of education							
Mother							
No schooling	2 (14.3)		4 (10.0)				
Primary	7 (50.0)		17 (42.5)		n.s.		
Secondary completed	4 (28.6)		11 (27.5)				
University	1 (7.1)		8 (20.0)				
Father							
No schooling	2(14.3)		6 (15.0)				
Primary	6 (42.9)		19 (47.5)		n.s.		
Secondary completed	5 (35.7)		12 (30.0)				
University	1 (7.1)		3 (7.5)				
Net income (€/mes)					n.s.		
<1,500	9 (60.0)		18 (46.2)				
1,500–2,500	4 (26.7)		16 (41.0)				
>2,500	2 (13.3)		5 (12.8)				
Pregnancy							
First pregnancy *	12 (80.0)		15 (37.5)		<0.01		6.6 (1.6–27.5)
BMI prepregnancy (kg/m^2^)		23.3 (20.7–25.9)		24.5 (22.9–26.1)		n.s.	
Folic acid periconceptional	2 (13.3)		19 (47.5)		<0.05		0.2 (0.1–0.8)
Weight gain (Kg) *						<0.05	0.8 (0.6–1.0)
PW 20–24		1.2 (−1.6-4.1)		3.5 (2.5–4.6)			
Legal and illegal drugs periconcepcional							
Maternal smoking (yes/no) * Cig/day	12 (80.0)	12.7 (5.1–20.3)	19 (47.5)	11.0 (6.5–15.5)	<0.05	n.s.	4.4 (1.1–18.0)
Paternal smoking (yes/no) * Cig/day	12 (80.0)	11.8 (7.4–16.2)	22 (55.0)	14.5 (10,0–18,9)	n.s.	n.s.	
Tobacco intrauterine (yes/no)	13 (86.7)		29 (72.5)		n.s.		
Alcohol intake mother (dichotomy = yes) g/day	11 (73.3)	11.8 (7.0–16.6)	26 (65.0)	11.6 (2.2–20.9)	n.s.	n.s.	
Number Binge drinke *	4 (26.6)	5.6 (0.4–11.7)	7 (17.5)	0.2 (0.1–0.3)		0.05	1.3 (0.8–2.0)
Alcohol intake father (dichotomy = yes) g/day	13 (86.7)	21.1 (15.2–26.9)	35 (87.5)	15.7 (7.2–23.1)	n.s.	n.s.	
Cannabis/marijuana **** ****							
Passive exposure (≥1 t/sem) *	10 (66.7)		7 (17.5)		<0.01		9.4 (2.4–36.3)
Maternal consumption							
(≥ 1t/week) *	5 (33.3)		2 (5.0)		<0.05		9.5 (1.6–56.4)
Medical ionizing radiation	3 (20.0)		2 (5.0)		n.s.		
Psychosocial factors							
Cohabitation time (months) Time before last period *		2.2 (−0.83–5.4)		82.5 (61.4–103.5)		<0.01	0.8 (0.8–1.0)
Cohabitation 1° term (if) *	9 (60.0)		35 (87.5)		<0.05		0.2 (0.1–0.8)
Paternal leave home 1° term *	3 (20.0)		0 (0)		<0.01		9.7 (0.9–102.6)
Physical abuse *	3 (20.0)		0 (0)		<0.01		9.7 (0.9–102.6)
Gender violence *	11 (73.3)		3 (7.5)		<0.01		33.9 (6.5–175.0)
Emotional stress “subjective” *	12 (80.0)		8 (20.0)		<0.01		16.0 (3.6–70.5)
Contraceptive use (any)	6 (42.9)		26 (65.0)		n.s		
Preservative use *	0 (0)		11 (27.5)		<0.05		0.7 (0.5–0.8)
Wanted pregnancy ****							
Woman *	3 (20.0)		26 (65.0)		<0.01		0.1 (0.1–0.5)
Man	8 (53.3)		26 (65.0)		n.s.		

PW = pregnancy week; t/week = times/week; d = day; * = variables with significant difference between cases and controls in variables analyzed. Gender violence defined as the sum of traumatic separation at first term, father imprisoned, physical or psychological abuse and/or anxiety attack related with psychological abuse (tested on clinic report). * = This means that the father have been reported for abuse. N.S. = non-significant.

The multivariate logistic regression model further highlights the periconceptional variable “gender violence” (OR:16.6; IC95% 2.7–101.7) and younger maternal age (OR:1.1; IC95% 1.0–1.3). The results suggest that both early maternal age and the occurrence of gender violence lead to the onset of the disease. 

## 4. Discussion

Results suggest that both early maternal age and living in a stressful environment related to maternal violence during periconceptional time are associated with the onset of the disease. To this point gender violence has been related to a greater risk of prematurity, heightened level of feto-maternal morbidity and mortality and low birth weight [30,35,36]. Our research shows an association of the gender violence with teratogenic outcomes. 

Early parental age is a known risk factor for gastroschisis [5,6,7,8,9,10,11,12,37]. High legal and illegal drugs consumption, as well as, dietary changes are most likely to occur among teenagers and young adults. In comparison to previous research studies, in this study there is a higher exposure to legal [38,39] and illegal [40,41,42] drugs in gastroschisis cases. 

Previous research relates low socioeconomic status to a greater risk of gastroschisis. Moreover, socioeconomic status was previously related to higher exposure rate to tobacco smoking, illicit drug use, a lower academic level, marginalized population and early and unplanned pregnancies [43]. Recently, gastroschisis was associated with the paternal role in pregnancy: on a biological level, as much as a social one. It has been suggested that the paternity change [26] and short parental cohabitation period [9] are both risk factors for gastroschisis due to an adverse effect on maternal immunity. In our research, we find that parental cohabitation time of cases is extremely short or non-existent. Most couples did not live together during the period of early pregnancy, and half of those who did live together, the father proceeded to leave the pregnant mother. For four fathers and in two cases, the mother is their second mate. Mothers of children with gastroschisis are significantly more often young women undergoing their first pregnancy, who, unlike their mates, were not seeking to have a child. The paternal role of some fathers of children born with gastroschisis was very dominant in the relationship, distorting the emotional framework and social relations of the mothers. The presence of physical abuse, anxiety triggered through problems in the relationship, the need to perform a tubal ligation as an alternative following the adolescents pregnancy, are all characteristic signs of strained household and circumstances. 

For the 15 cases, the average maternal age was 21.6 (95%IC 18.8–24.3). There is evidence to suggest that the mothers less than 20 years of age have an increased risk for gastroschisis [5,6,7,8,9,10]. However, the pregnant women aged 20 to 24 years, the odds were 1.9 (95% CI 0.7–5.0) [6]. Also, there is difficulty in differentiating between the etiological factors leading to gastroschisis, since young maternal age may be a risk factor for the development of birth defects, but lifestyle choices such as drug abuse and physical abuse appear to be involved as well. Adolescent women (in this study 5/15 mothers of cases are younger than 19 years of age) have an immature perception of the risks of tobacco smoking, alcohol intake, illegal drugs use and total well-being. With a certain frequency, adolescent pregnancy is associated with gender violence and absence of sexual and reproductive rights [44]. Young women and men are less inclined to practice safe sex, and often lack a stable, long-term partnership. Even the fact that men surveyed in this study were looking for women who were barely teenagers to have their children, could be considered gender-based violence. 

The adolescent show heightened cardiovascular reactivity [45]. Pregnancy during adolescence exerts lasting pro-atherogenic effects independent of weight gain [46]. The prenatal psychosocial stress has been found to be associated with the offspring’s cardiovascular-metabolic risk [47]. A potentially involved physiological system is the fetal hypothalamus-pituitary-adrenal (HPA) axis, responsible for stress-regulation and particularly susceptible to programming during fetal life [47]. On the other hand, the first trimester seems to be particularly important as the HPA-axis of the fetus is most vulnerable for dysregulation during this period. The teratogenic action mechanisms of domestic violence could be explained by the maternal-placental-fetal neuroendocrine parameters that they are significantly associated with features of maternal psychosocial functioning in pregnancy [48]. 

We will mention some limitations of this work: first, the reduced number of cases derived from the low prevalence of gastroschisis and second the possibility that some cases could not have been registered or had been incorrectly classified. We have developed a centralized public regional network with an exhaustive search with strict inclusion–exclusion criteria that increases the consistency of our data. All suspected cases of prenatal diagnosis were confirmed postnatally. We trust that sample size of this case-control study will grow with time and collaboration of the Pediatric Surgery Services of Spain and Europe. Thirdly, recall bias should be considered in observational research. Most of the interviews were conducted during pregnancy by the trained staff of our environmental health and risk team. However, since the possible association of gastroschisis is not regarded by the general public or the medical community as possibly related to gender violence, it is unlikely that a bias might have been introduced. Forth, the interaction between gender violence and adolescent pregnancy. Adolescent pregnancy is currently a relevant problem and is an issue of major social concern. The etiology includes biological, social and cultural factors, but not gender violence [49]. We have investigated the existence of interaction and/or confounding nature between variables by constructing and comparing logistic regression models. An additional limitation to our study is the measurement of domestic violence. We defined the study variables of gender violence that include very objective aspects, such as couples who have experienced traumatic break ups during the first weeks of pregnancy, imprisoned partners who have a background of abuse, abuse itself (verified with complaint) and/or an anxiety attack (documented clinic reports) (see Table 3). In this ongoing project, we incorporated internationally validated surveys of gender violence that allowed us to compare our results with other groups [31]. Conflicting variables have been controlled by the meticulous development of the ‘green sheet’ questionnaire. Interactions with dietary factors [17,18,19,20,21], infections [21,50] and drug intake like ibuprofen, aspirin, silicates and nasal descongestants [15] have also been taken into account. In order to guarantee maternal safety and the reliability of data, the couples were interviewed separately. 

While scientific evidence is increasing, an important progression of this research would be to examine gene-environment interactions [51] of the couple through a gender perspective. To prevent malformations and birth-defects such as gastroschisis, a much broader approach is necessary for: (a) Prevention of adolescent pregnancy through a gender perspective and co-responsibility between young men and women for contraceptive use; (b) Include the parent in fertility and pregnancy programs, involving the father in carrying out activities and receiving recommendations; (c) Finally, to address gastroschisis consultation with a gender perspective in order to assure adolescent maternal safety as a necessary factor to help improve the quality of life of children with gastroschisis, improve the quality of the relationship between the parents and plan better future pregnancies. 

## 5. Conclusions

The violence against pregnant women is associated with birth defects, and should be studied in more depth as cause-effect teratogenic. Although additional research is clearly needed, our findings have potentially significant implications for current public health programs. Screening for ongoing gender violence should be incorporated into routine prenatal care to identify women at risk of complications and preventable birth defects such as gastroschisis. A comprehensive and careful analysis of environmental exposures used to detect psychosocial risk factors, including gender-based violence, are important to the improved healthcare quality, integrity, and for insuring the health and safety of the pregnant mother and the fetus. 

## Members of the Translational Research Group on Gastroschisis

Hospital Clínico Universitario Virgen de la Arrixaca, Murcia, Spain. Servicio de Pediatría: M. Sánchez-Solís, A. Brea Lamas, J.A. Ortega-García, A. Cánovas-Conesa, V. Gomariz-Peñalver, M.F. Sánchez-Sauco; M. Martínez Aroca, E. Guillén Navarro, D. Jaimes-Vega. Servicio de Cirugía Pediátrica: J.I. Ruiz-Jiménez; M.J. Aranda-García, A. Trujillo-Ascanio, M. Fernández-Ibieta; Unidad de Medicina Fetal, Servicio de Obstetricia y Ginecología: J.L. Delgado Marín, C. de Paco Matallana, A. Arteaga Moreno, A. Nieto Saiz. Coordinación Regional de Drogodependencias Murcia: J. Jiménez Roset, A.B. Villar Lorenzo, C. Puerta Ortuño. Universidad Politécnica de Cartagena, Cartagena, Spain: F. López Hernández. Georgetown University Medical Center, Washington DC, USA: Offie P. Soldin. 
